# Apatite-like calcium phosphate deposition and physicochemical properties of an aspartic acid–functionalized hydraulic cement

**DOI:** 10.1007/s00784-026-07196-w

**Published:** 2026-10-03

**Authors:** Elias Daniel Covas Rodrigues, Victor Miguel Polizeli, Júlia Tavares de Sousa, Larissa Fernanda Pereira, Regina Guenka Palma-Dibb, Larissa Moreira Spinola de Castro-Raucci, Marcelle Danelon, Walter Raucci-Neto

**Affiliations:** 1 School of Dentistry of Ribeirão Preto (FORP/USP), Department of Restorative Dentistry, University of São Paulo, Ribeirão Preto, SP Brazil; 2 University of Ribeirão Preto, Ribeirão Preto, SP Brazil; 3 Universitätsklinikum Carl Gustav Carus an der Technischen Universität Dresden, Dresden, Germany

**Keywords:** Aspartic acid, Calcium silicate cement, Mineral trioxide aggregate, Biomineralization, Physicochemical properties, Bioactive dental materials

## Abstract

**Objectives:**

This study evaluated the effects of aspartic acid modification on the physicochemical properties and phosphate-associated surface response of a hydraulic calcium silicate cement.

**Materials and methods:**

White mineral trioxide aggregate (MTA) was mixed using 0%, 25%, 50%, 75%, or 100% (v/v) of a 4 g/L aspartic acid stock solution. Final setting time, solubility, pH, and Ca²⁺ concentration in the immersion solution were evaluated after seven days in deionized water (*n* = 10). Surface roughness, morphology, elemental composition, and phosphate- and carbonate-associated spectral features were assessed after 30 days of storage in deionized water or phosphate-buffered saline (PBS). The Ap/C spectral ratio was calculated from the integrated ATR-FTIR band areas. Data were analyzed using one- or two-way ANOVA followed by Tukey’s test (α = 0.05).

**Results:**

The 25% formulation showed the shortest setting time, whereas the 100% formulation showed the longest (*p* < 0.001). All aspartic acid-containing formulations exhibited greater solubility and lower Ca²⁺ concentrations than the control (*p* < 0.05), and the 50%, 75%, and 100% formulations exceeded the recommended solubility limit. The 25% formulation had the lowest pH, whereas the control, 75%, and 100% formulations maintained similar alkaline values. Surface roughness was lowest for the 25% formulation and highest for the 100% formulation, with no significant effect of storage medium. EDS detected negligible surface phosphorus after storage in deionized water but marked phosphorus content after PBS storage. Ap/C analysis showed significant main effects of cement formulation and storage medium, without a significant interaction. The 25%, 50%, and 75% formulations exhibited higher marginal Ap/C ratios than the 0% and 100% formulations.

**Conclusions:**

Aspartic acid produced non-linear and formulation-dependent changes in the evaluated properties. Higher Ap/C ratios reflected differences in relative spectral composition but did not confirm increased apatite formation or a crystalline apatite phase. Ca–P-rich surface deposition depended on phosphate supplied by the storage medium, and no formulation improved all evaluated outcomes.

## Introduction

Biomimetic strategies have been extensively investigated to enhance dentin mineralization, particularly through the use of molecules capable of mimicking the function of non-collagenous proteins involved in the transport and stabilization of amorphous calcium phosphate (ACP), the precursor of hydroxyapatite [1–3]. These biomimetic approaches aim to reproduce physiological mineralization mechanisms and have been explored as promising strategies for the repair and regeneration of mineralized dental tissues [4, 5].

Among the molecules investigated for this purpose, aspartic acid has attracted attention due to its ability to regulate mineral nucleation and growth within collagen matrices. As a naturally occurring amino acid, it plays an important role in mineralized tissues, where its negatively charged carboxylate groups interact with calcium (Ca²⁺) and phosphate (PO₄³⁻) ions, promoting the formation and stabilization of mineral precursors [3, 6].

In this context, aspartic acid has been shown to contribute to the nucleation and stabilization of amorphous calcium phosphate (ACP), facilitating its controlled transformation into hydroxyapatite. Although ACP represents a crucial intermediate phase in the mineralization process, its intrinsic instability may lead to premature dissolution [7]. The presence of aspartic acid and other acidic biomolecules can reduce this instability by stabilizing ACP nanoclusters and regulating their transformation kinetics, promoting a more organized mineralization pathway and preventing uncontrolled crystal growth [8, 9]. This mechanism is particularly relevant in biomimetic dentin mineralization, as stabilized ACP precursors are able to infiltrate the exposed collagen matrix and subsequently crystallize into hydroxyapatite, contributing to intrafibrillar mineralization, structural reinforcement, and tissue regeneration [6, 10–12].

Previous studies have demonstrated that aspartic acid can facilitate the remineralization of demineralized dentin by promoting mineral deposition within the collagen matrix and by modulating the balance between mineral dissolution and precipitation [3]. Despite these promising findings, the potential application of aspartic acid in calcium silicate–based hydraulic cements used for direct pulp capping procedures, particularly in the context of partially demineralized dentin substrates, remains poorly investigated.

Calcium silicate–based cements, such as mineral trioxide aggregate (MTA), are widely used in direct pulp capping due to their bioactivity and ability to promote mineralized tissue formation. This bioactivity is closely associated with Ca²⁺ concentration in the immersion solution and subsequent apatite-like Ca–P deposition at the material–tissue interface. However, despite these favorable properties, the mineralization process induced by MTA is largely dependent on passive ionic interactions and may lack the level of structural organization observed in physiological biomineralization [13, 14]. In this context, strategies aimed at actively modulating mineral nucleation and growth represent an important and still underexplored field.

Although biomimetic molecules such as aspartic acid have demonstrated the ability to regulate ACP stabilization and collagen-guided mineralization, their application has been predominantly investigated in dentin remineralization models [1, 15]. There is a clear lack of evidence regarding the incorporation of such molecules into calcium silicate–based hydraulic cements, particularly concerning their potential to influence the hydration reaction, calcium ion dynamics, and the formation of biomimetic mineral phases.

Compared with unmodified MTA, the proposed added value of aspartic acid lies in the introduction of carboxylate groups capable of interacting with Ca²⁺ and potentially regulating mineral precursor stabilization and surface mineral-related processes. Therefore, functionalization was not intended merely to increase calcium release, but to investigate whether these molecular interactions could modify cement hydration and the relative phosphate-associated surface response. Whether these effects could occur without compromising the physicochemical stability of the cement remained uncertain and formed the basis for the present comparison.

Therefore, this study aimed to investigate whether the functionalization of a calcium silicate–based hydraulic cement with aspartic acid could modulate its physicochemical properties and biomineralization behavior. Specifically, the study evaluated the effect of different proportions of an aspartic acid solution used as the mixing liquid on parameters related to hydration, Ca²⁺ concentration in the immersion solution, and apatite-like Ca–P deposition. The null hypothesis tested was that the incorporation of aspartic acid would not influence the physicochemical properties or the biomineralization potential of the material, regardless of the concentration used.

## Materials and methods

### Experimental design and ethical considerations

This study did not involve human participants or live animals; therefore, it was exempt from evaluation by an institutional ethics committee.

The experimental factor was the proportion of a 4 g/L aspartic acid solution used as the mixing liquid for mineral trioxide aggregate (MTA), evaluated at five levels: 0% (control, 100% deionized water), 25%, 50%, 75%, and 100% (v/v). For biomineralization and surface characterization, a two-factor design was adopted, considering the proportion of aspartic acid and the storage medium (deionized water or PBS).

Ten independent specimens were used per experimental group for each analysis (*n* = 10), as determined by a sample size calculation performed using G*Power (version 3.1; α = 0.05; power = 0.969). For surface and chemical characterization, ten specimens were evaluated for each combination of aspartic acid proportion and storage medium. Confocal laser scanning microscopy (CLSM) analyses followed previously established protocols [16].

### Materials and mixing procedures

White mineral trioxide aggregate (MTA; Angelus, Londrina, PR, Brazil) was used. The crystalline phases previously reported for this material by Setbon et al. (2014) [17] include Ca₃SiO₅, Ca₂SiO₄, Ca₃Al₂O₆, CaO, SiO₂, and Bi₂O₃, as summarized in Table 1. The cement was mixed at a powder-to-liquid ratio of 1 g/0.33 mL [18].

**Table 1:** Chemical composition of white mineral trioxide aggregate (MTA; Angelus)

Component	Composition
MTA Angelus – powder	Tricalcium silicate, Dicalcium silicate, Tricalcium aluminate, Calcium oxide, Silicon oxide, Bismuth oxide
MTA Angelus – liquid	Distilled water

A 4 g/L L-aspartic acid stock solution, corresponding to approximately 30 mmol/L, was prepared by dissolving 4 g of L-aspartic acid in 1 L of deionized water. This concentration was selected based on the findings of Gómez-Morales et al. (2011), who demonstrated that 30 mmol/L L-aspartic acid could modify calcium phosphate precipitation and phase selection [18]. The stock solution was used as the highest experimental concentration and was mixed with deionized water at the predefined volumetric proportions to evaluate concentration-dependent effects. Because an unadjusted aqueous solution of L-aspartic acid is acidic and could interfere with MTA hydration, its pH was adjusted to 9.5 by the controlled addition of 10 M NaOH. This adjustment provided alkaline mixing conditions and ensured that the carboxyl groups of aspartic acid remained predominantly deprotonated, favoring their availability for interaction with calcium ions. The final mixing liquid was obtained by diluting the aspartic acid solution (4 g/L) in deionized water to achieve the following proportions: 0%, 25%, 50%, 75%, and 100% (v/v).

### Setting time, solubility, pH, and Ca²⁺ release analysis

Setting time was assessed using specimens prepared with cylindrical stainless-steel molds. From 120 ± 10 s after the start of mixing, samples were stored in an incubator until completion of the test.

Initial setting time was determined using a Gillmore needle (400 g load; 1.0 mm tip diameter). The first measurement was performed at 150 ± 10 s after mixing, with subsequent applications at 60-second intervals. Final setting time was defined as the point at which the needle no longer produced visible indentation on the material surface.

Solubility specimens were prepared using circular Teflon molds (7.75 mm internal diameter and 1.5 mm thickness). The molds were placed on glass slides covered with cellophane and filled with the test cement. A nylon thread was embedded in each specimen, and a second covered glass slide was applied under manual pressure to ensure complete surface contact. Ten specimens were prepared per group.

After setting, the specimens were removed from the molds and weighed to determine their initial mass (m_i_). Each specimen was suspended by its nylon thread in a separate container with 7.5 mL of distilled deionized water and maintained at 37 °C for seven days without renewal of the immersion medium. At the end of this period, the specimens were removed, rinsed, dried in a desiccator containing 98% sulfuric acid for 24 h, and reweighed to determine their final mass (mᶠ). Solubility was expressed as percentage mass loss using the following formula: dm% = 100 × (m_i_ − mᶠ)/m_i_.

The ten individual immersion solutions, each with an original volume of 7.5 mL, constituted independent experimental replicates for the pH and Ca²⁺ release analyses. The pH was measured directly in each solution once, immediately after completion of the seven-day immersion period, using a calibrated multiparameter meter (AK151; AKSO). No pH measurements were performed before immersion or at intermediate time points. Thus, the recorded values represented the cumulative alkalinizing effect of the cements during the immersion period.

After pH measurement, the Ca²⁺ concentration was determined in aliquots collected from the same immersion solutions using flame atomic absorption spectrometry (SpectrAA 200; Varian, Melbourne, Australia) at a wavelength of 422.7 nm, with a calcium-specific hollow cathode lamp as the radiation source. A 1,000 µg/mL Ca²⁺ stock standard solution (Merck, Darmstadt, Germany) was used to prepare calibration standards of 1.0, 2.0, 3.0, 4.0, and 5.0 µg/mL. Before analysis, each aliquot was diluted 30-fold with distilled deionized water (1:30, v/v; one volume of sample and 29 volumes of water) to obtain concentrations within the calibration range. Only measurements within the 1.0–5.0 µg/mL interval were accepted. The Ca²⁺ concentration in the original immersion solution was calculated by multiplying the concentration measured in the diluted aliquot by the dilution factor of 30. Results were expressed as µg/mL of the original immersion solution and were not additionally normalized by specimen mass or surface area because all specimens had standardized dimensions and were individually immersed in the same solution volume (7.5 mL). The pH and Ca²⁺ measurements were each performed in technical triplicate for every immersion solution, and the mean of the three readings was assigned to the corresponding specimen. Thus, the ten independently prepared specimens and their respective immersion solutions resulted in an experimental sample size of *n* = 10 per group for statistical analysis.

### SEM and CLSM surface characterization

Cylindrical specimens (4 mm in diameter and 2 mm in height) were prepared and stored for 30 days in either deionized water or phosphate-buffered saline. After storage, the specimens were dried in a silica-containing desiccator for 24 h.

Surface morphology was analyzed by scanning electron microscopy (SEM) using a ZEISS EVO 50 microscope (Zeiss, Germany) operated at 20 kV. Before analysis, the specimens were sputter-coated with a gold–palladium alloy. Micrographs were acquired at 1000× for qualitative comparison of surface organization and the distribution of superficial deposits among the experimental groups. Detailed characterization of nanoscale structures or the morphology of individual deposits was beyond the scope of this analysis.

Surface roughness and topography were evaluated by CLSM using a LEXT OLS 400 system (Olympus, USA). The specimens were mounted on glass slides using a parallelometer and an adhesive compound (Pritt, Henkel) to ensure that the analyzed surface remained parallel to the slide and perpendicular to the microscope objective, thereby standardizing specimen positioning. Three-dimensional images of the central region of each specimen were obtained using a 10× objective. The system, equipped with a 405 nm semiconductor laser, enabled three-dimensional surface acquisition. A standardized central area was selected for each specimen, and surface roughness was quantified using the Sa parameter (arithmetical mean surface roughness), according to ISO 25,178, with values expressed in micrometers (µm). Corresponding topographic images were also recorded for each specimen.

### Chemical characterization by EDS and FTIR

Chemical surface characterization after 30 days of immersion in deionized water and phosphate-buffered saline (PBS) was performed using Fourier-transform infrared spectroscopy with attenuated total reflectance (ATR-FTIR) and energy-dispersive X-ray spectroscopy (EDS) coupled to scanning electron microscopy. Phosphorus was quantified under both storage conditions because deionized water served as a phosphate-free control. This comparison allowed the phosphorus detected after PBS storage to be attributed to phosphate supplied by the external medium rather than to the original cement composition or experimental contamination.

For EDS analysis, specimens were mounted on aluminum stubs, sputter-coated with a thin gold layer, and examined under a scanning electron microscope operated at 20 kV. Elemental composition was determined by point analysis on representative surface areas, and the relative weight percentages (wt%) of key elements, particularly phosphorus (P), calcium (Ca), and silicon (Si), were recorded. This approach allowed the assessment of surface mineral deposition and the detection of Ca–P-rich surface regions formed after immersion in different media. Data were expressed as mean ± standard deviation for each group.

For ATR-FTIR analysis, specimens were dried in a silica-containing desiccator for 24 h and analyzed using an IRSpirit-X (Shimadzu, Kyoto, Japan) with a spectral resolution of 4 cm⁻¹ and 32 scans per sample. Spectra were processed using Origin 2020 (OriginLab, USA) and restricted to the 500–1800 cm⁻¹ region, corresponding to vibrational modes associated with hydrated calcium silicate phases (C–S–H), phosphate (PO₄³⁻), and carbonate (CO₃²⁻). For quantitative assessment of apatite-like phase formation, specific spectral windows were defined based on previously established criteria for phosphate and calcium carbonate identification [19, 20]. Phosphate-related contributions were quantified by integrating the absorbance areas of the PO₄³⁻ ν₁ band (~ 960 cm⁻¹) and the PO₄³⁻ ν₄ doublet (~ 563 and 602 cm⁻¹), whereas carbonate contributions were determined from the ν₃ CO₃²⁻ band (1415–1465 cm⁻¹). Band areas were calculated by trapezoidal numerical integration within fixed wavenumber intervals, and the apatite-to-carbonate ratio (Ap/C) was calculated as (Area₉₆₀ + Area₅₆₃ + Area₆₀₂) / Area₁₄₁₅–₁₄₆₅. Comparative analysis of the spectral profiles (500–1800 cm⁻¹) was then performed across the experimental groups.

### Statistical analysis

Statistical analyses were performed using Jamovi (The Jamovi Project, version 2.5.0). Data normality and homogeneity of variances were assessed using the Shapiro–Wilk and Levene tests, respectively. Statistical tests were selected according to the experimental design of each outcome. Data involving cement formulation as the only independent factor were analyzed using one-way ANOVA. Surface roughness and the Ap/C ratio were analyzed using two-way ANOVA, with cement formulation and storage medium as the independent factors, including their interaction. When significant differences were detected, multiple comparisons were performed using Tukey’s post hoc test. The significance level was set at 5%. Morphological analyses were performed qualitatively.

## Results

### Setting time, solubility, pH, and Ca²⁺ release

The physicochemical properties evaluated, including setting time, solubility, pH, and Ca²⁺ release, are summarized in Table 2. Setting time was reduced at 25% and 50% compared with higher concentrations, with the shortest values observed at 25%. the 25% formulation differed from 0%, 75%, and 100% (*p* ≤ 0.038), but not from 50% (*p* = 0.252). Similarly, 50% showed lower values than 75% and 100% (*p* ≤ 0.018), with no difference from 0% (*p* = 0.901). The 100% concentration exhibited the longest setting time, differing from all other groups (*p* < 0.001), whereas no difference was observed between 0% and 75% (*p* > 0.05).

The control group (0%) exhibited significantly lower solubility than all other groups (*p* < 0.001). The 25% group also presented significantly lower values compared with the 50%, 75%, and 100% groups (*p* < 0.001). No statistically significant differences were observed among the 50%, 75%, and 100% groups (*p* > 0.05), indicating similar solubility behavior at higher aspartic acid proportions.

The 25% group exhibited significantly lower pH values compared with all other groups (*p* < 0.001). The 50% group also presented significantly lower pH than the 75% (*p* < 0.001) and 100% (*p* < 0.001) groups. No statistically significant differences were observed among the 0%, 75%, and 100% groups (*p* > 0.05), indicating similar alkaline behavior among these formulations.

The 0% group exhibited significantly higher Ca²⁺ release than all other experimental groups (*p* < 0.05). No statistically significant differences were observed between the 25% and 50% groups (*p* = 0.416), nor between the 75% and 100% groups (*p* = 0.310). Additionally, the 25% and 50% groups presented significantly lower Ca²⁺ release compared with the 75% and 100% groups (*p* < 0.01).

**Table 2:** Mean values and standard deviations of setting time (minutes), solubility, pH, and calcium release of the experimental cements in deionized water and PBS

Groups	Setting time (min)	Solubility (%)	pH	Ca²⁺(µg/mL)
0%	21.5^BC^ ± 0.707	-2.58ᴬ ± 0.654	11.10^C^ ± 0.158	92.1^C^ ± 16.18
25%	19.5^A^ ± 1.354	1.88ᴮ ± 0.555	8.31ᴬ ± 0.524	46.1ᴬ ± 5.14
50%	20.9^AB^ ± 0.876	6.80^C^ ± 0.334	9.35ᴮ ± 0.866	51.7ᴬ ± 8.39
75%	23.1^C^ ± 1.101	7.62^C^ ± 0.692	11.16^C^ ± 0.119	65.8ᴮ ± 7.68
100%	28.4^D^ ± 2.675	6.70^C^ ± 1.274	11.32^C^ ± 0.169	71.9ᴮ ± 6.01

Different superscript letters indicate statistically significant differences among groups within each evaluated parameter (*p* < 0.05), whereas identical letters indicate no statistically significant difference (*p* > 0.05).

### Surface morphology and roughness (SEM and CLSM)

CLSM analysis showed a significant main effect of cement formulation on surface roughness (*p* < 0.001). Neither the main effect of storage medium (*p* = 0.103) nor the formulation-by-storage medium interaction (*p* = 0.093) was significant. These findings indicate that Sa values differed among cement formulations but not between storage media under the conditions evaluated.

The 25% group exhibited significantly lower roughness than the control (0%) (*p* = 0.028), as well as the 75% (*p* = 0.002) and 100% (*p* < 0.001) groups, with no significant difference compared to the 50% group (*p* > 0.05).

The 100% group presented the highest mean roughness values, differing significantly from the 0% (*p* = 0.003), 25% (*p* < 0.001), and 50% (*p* < 0.001) groups. Significant differences were also observed between the 50% and 75% groups (*p* = 0.021), and between the 75% and 100% groups (*p* = 0.041), indicating increased roughness at higher proportion of the aspartic acid stock solutions. Overall, the lower aspartic acid proportion (25%) reduced surface roughness, whereas higher proportions (75% and 100%) were associated with increased surface irregularity. Mean values and standard deviations are presented in Table 3.

**Table 3:** Mean values and standard deviations of surface roughness (Sa, µm) for the evaluated groups

0%	Deionized Water	PBS	Mean
	4.724 ± 1.754	5.755 ± 2.716	5.240^BC^±2.222
**25%**	3.100 ± 0.275	2.771 ± 0.701	2.936^A^±0.531
**50%**	4.008 ± 2.883	3.127 ± 1.375	3.567^AB^±2.179
**75%**	5.447 ± 0.659	6.445 ± 1.240	5.946^C^±1.073
**100%**	6.581 ± 0.743	9.681 ± 1.965	8.131^D^±2.151
**Mean**	4.772^A^ ± 1.333	5.556^A^ ± 2.806	

The same superscript letter indicates no statistically significant difference.

**Fig. 1:**
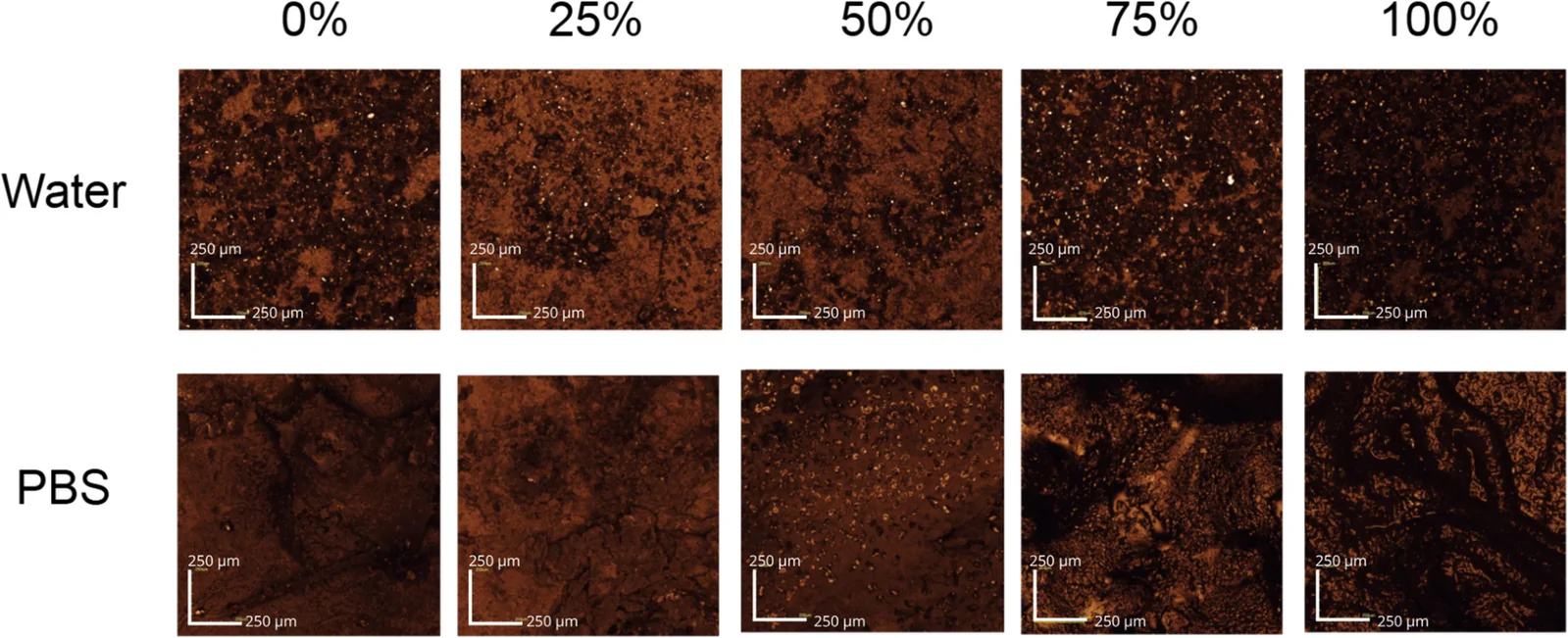
Representative confocal laser scanning microscopy images of the experimental groups after 30 days of storage in water or PBS

Complementary to the quantitative analysis, morphological evaluation by confocal laser scanning microscopy enabled visualization of topographical differences among the experimental groups after 30 days of storage. The images revealed distinct surface irregularity patterns, corroborating the findings obtained from surface roughness (Sa) analysis. Representative micrographs for each group, in both water and PBS storage media, are presented in Fig. 1.

**Fig. 2:**
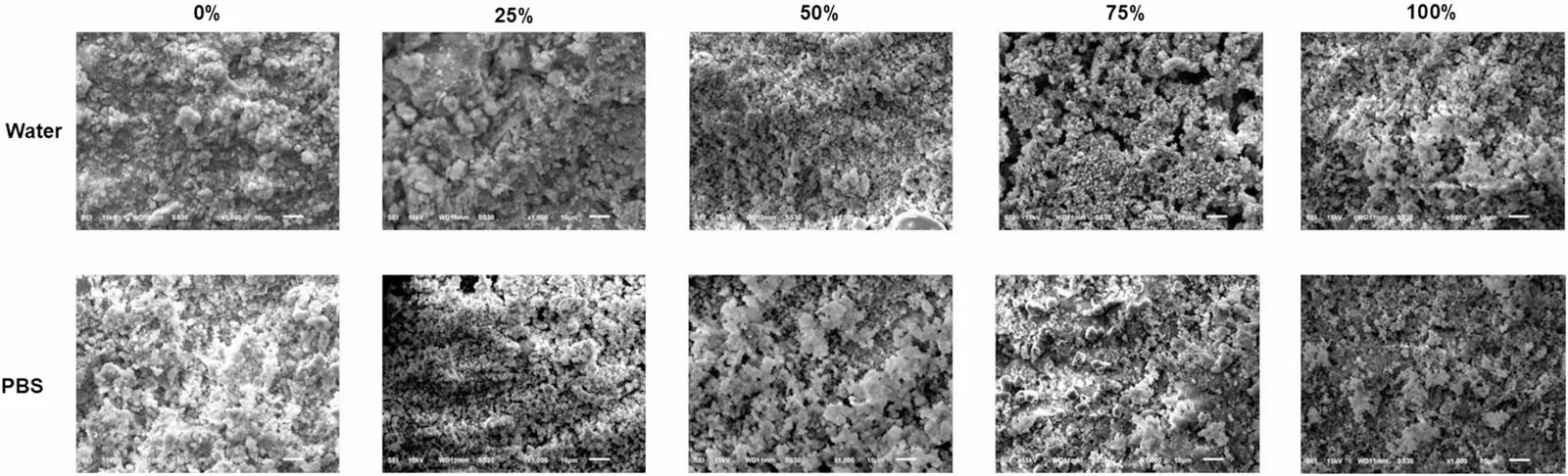
Representative SEM micrographs of each experimental group after storage in water or PBS, obtained at 1000× magnification

SEM analysis showed qualitatively similar surface morphologies among the experimental groups, regardless of the proportion of the aspartic acid stock solution or storage medium. The specimens generally exhibited an irregular and granular surface composed of particle agglomerates, globular or irregularly shaped deposits, and occasional pores. No consistent concentration-dependent pattern in surface organization or deposit morphology was identified. Likewise, comparison between specimens stored in deionized water and PBS did not reveal clearly distinguishable morphological patterns by SEM. Because no morphometric analysis of the surface deposits was performed, the SEM findings were interpreted descriptively. Therefore, the main differences between the storage media were related to the chemical composition of the surface deposits, as demonstrated by EDS and FTIR, rather than to readily distinguishable morphological changes. Representative micrographs of each group after storage in deionized water and PBS are presented in Fig. 2.

### Chemical characterization by EDS and ATR-FTIR

EDS analysis (Table 4) showed negligible phosphorus in specimens stored in deionized water across all concentrations (0–100%), with mean values ranging from 0.02 ± 0.03 to 0.05 ± 0.04 wt%, indicating the absence of surface phosphorus content regardless of the mixing condition. In contrast, specimens immersed in PBS exhibited marked phosphorus incorporation at all concentrations, with mean values ranging from 9.17 ± 3.63 to 12.25 ± 1.98 wt%. Phosphorus incorporation was relatively consistent at 0% and 100%, while greater variability was observed at intermediate concentrations (25–75%). Calcium remained a major constituent in all groups, whereas silicon tended to be higher in specimens stored in deionized water and lower in those immersed in PBS, consistent with the formation of Ca–P-rich surface deposits.

**Table 4:** Elemental composition (wt%) obtained by EDS of specimens prepared with different concentrations of the experimental solution and stored in deionized water or PBS

Concentration (%)	Medium	*P* (wt%)	Ca (wt%)	Si (wt%)
0%	Water	0.02 ± 0.03	36.14 ± 3.70	2.04 ± 1.25
	PBS	12.25 ± 1.98	35.24 ± 0.81	0.45 ± 0.62
25%	Water	0.04 ± 0.03	33.09 ± 4.04	3.81 ± 1.77
	PBS	9.17 ± 3.63	35.09 ± 0.54	1.14 ± 1.18
50%	Water	0.05 ± 0.04	37.18 ± 2.30	2.13 ± 0.58
	PBS	9.72 ± 5.26	34.92 ± 1.34	0.55 ± 0.63
75%	Water	0.04 ± 0.02	35.44 ± 3.59	1.89 ± 1.63
	PBS	9.23 ± 4.98	37.05 ± 1.26	0.47 ± 0.29
100%	Water	0.05 ± 0.04	32.47 ± 1.66	2.80 ± 0.38
	PBS	11.62 ± 1.00	37.08 ± 1.04	0.64 ± 0.60

Data are presented as mean ± standard deviation.

**Fig. 3:**
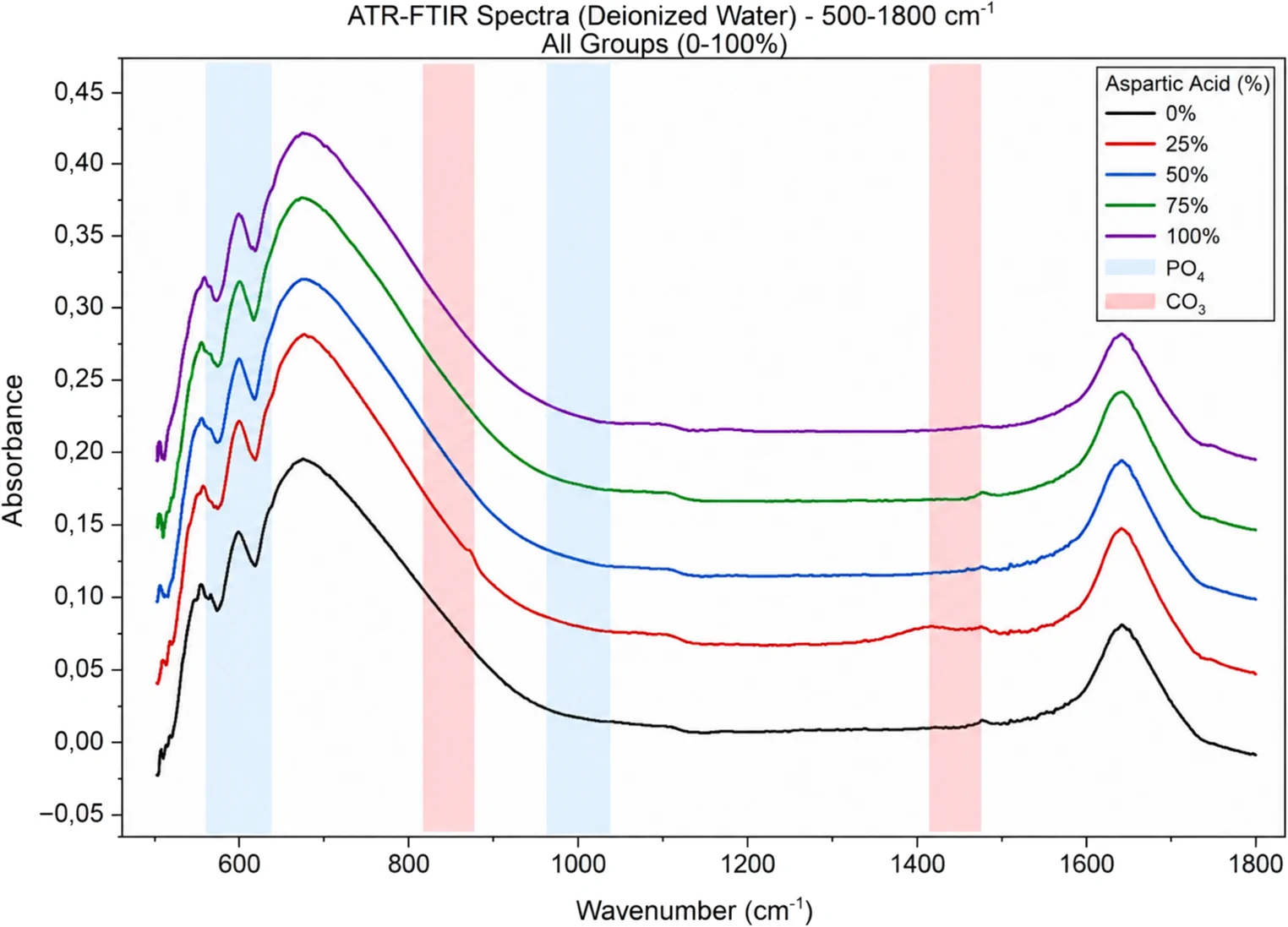
Mean ATR-FTIR spectra of the 0%, 25%, 50%, 75%, and 100% groups after 30 days of storage in deionized water (*n* = 5 per group). Spectra are displayed with constant vertical offsets for visual clarity. The offsets are graphical only and do not represent differences in absolute absorbance. Quantitative analyses were performed using the original unshifted spectra. Blue and red shaded regions indicate phosphate- and carbonate-associated bands, respectively

**Fig. 4:**
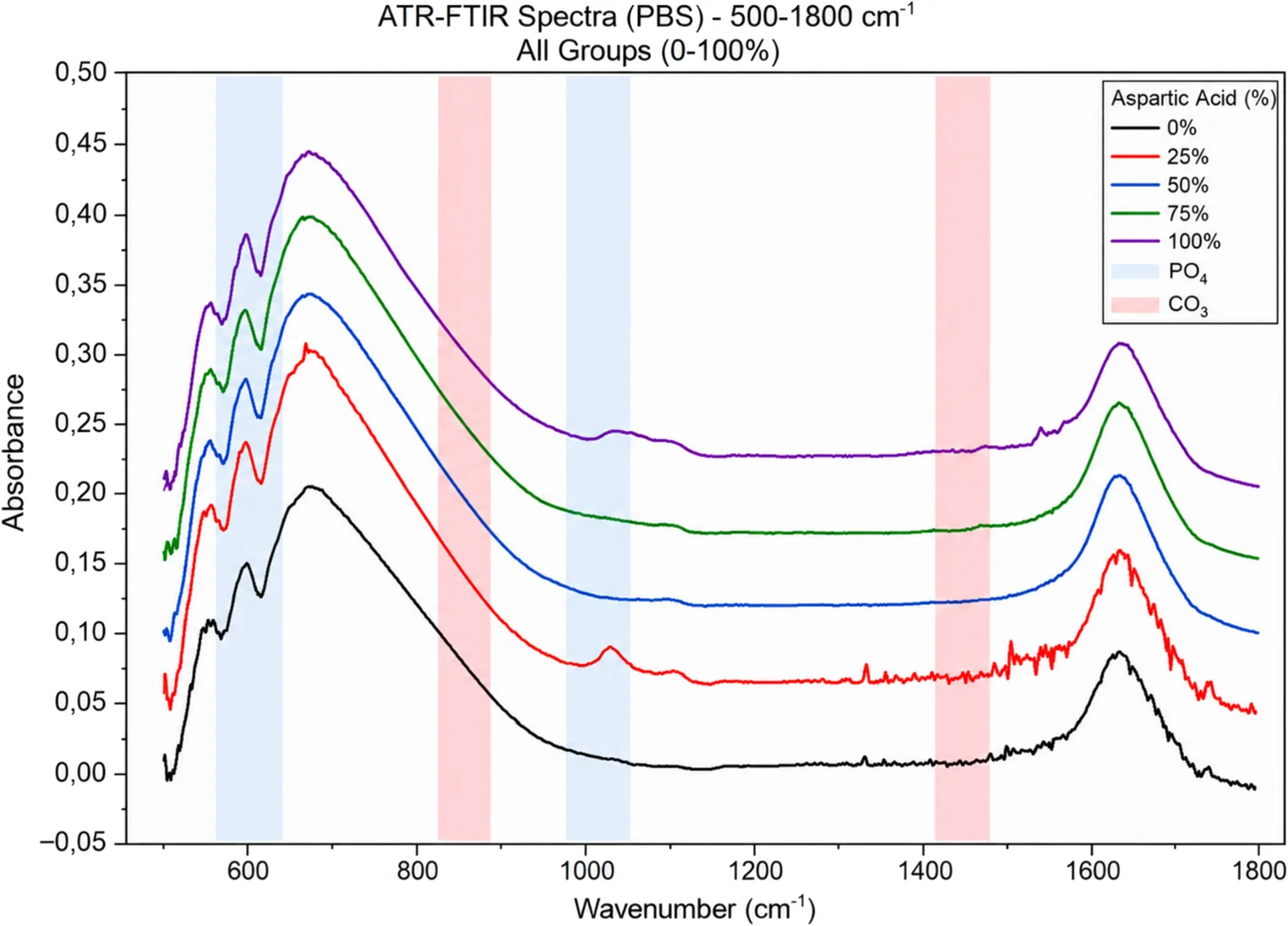
Mean ATR-FTIR spectra of the 0%, 25%, 50%, 75%, and 100% groups after 30 days of storage in PBS (*n* = 5 per group). Spectra are displayed with constant vertical offsets for visual clarity. The offsets are graphical only and do not represent differences in absolute absorbance. Quantitative analyses were performed using the original unshifted spectra. Blue and red shaded regions indicate phosphate- and carbonate-associated bands, respectively

Figures 3 and 4 show the mean ATR-FTIR spectra of the cements mixed using different proportions of the aspartic acid stock solution after 30 days of storage in deionized water and PBS, respectively. Visual comparison of the spectra allowed qualitative assessment of changes in the intensity of vibrational bands associated with mineral phases, providing insight into the material’s chemical behavior under different storage conditions.

For specimens stored in deionized water, differences in the intensity of phosphate-associated spectral regions were observed among the experimental groups, following the order 25% > 75% > 100% > 50% > 0%. The control group showed the lowest absorbance, whereas the 25% formulation showed the highest intensity in these spectral regions. However, because EDS detected negligible phosphorus in specimens stored in deionized water, these variations were interpreted as differences in phosphate-associated FTIR signals rather than evidence of effective surface phosphorus content or increased Ca–P deposition.

For specimens stored in PBS, the decreasing order of phosphate band intensity was: 25% > 50% > 75% > 100% > 0%. As observed for deionized water, the 25% group exhibited the highest absorbance values, while the control group showed the lowest. However, a more gradual numerical distribution of phosphate-associated band intensities was observed among the intermediate formulations stored in PBS.

The apatite-to-carbonate ratio (Ap/C) analysis showed significant main effects of cement formulation (*p* < 0.001) and storage medium (*p* = 0.003), with no significant interaction between the factors (*p* = 0.100). Tukey’s post hoc comparisons of the marginal means indicated that the 0% group had lower Ap/C values than the 25%, 50%, and 75% groups (*p* ≤ 0.002), but did not differ from the 100% group (*p* = 0.962). The 100% group also exhibited lower values than the 25%, 50%, and 75% groups (*p* ≤ 0.010), with no significant differences among the intermediate concentrations (*p* > 0.05). When averaged across cement formulations, specimens stored in PBS exhibited higher marginal Ap/C values than those stored in deionized water (6.00 ± 0.624 and 5.51 ± 0.904, respectively; *p* = 0.003). Although the 50% group showed a numerically higher mean in deionized water than in PBS, this within-group difference was not statistically significant (*p* = 0.999). Overall, Ap/C values were higher at intermediate concentrations (25–75%) than at 0% and 100% (Table 5).

**Table 5:** Mean apatite-to-carbonate ratios (Ap/C) of cements modified with different concentrations of aspartic acid after 30 days of immersion in water or PBS, calculated from ATR-FTIR integrated band areas (mean ± standard deviation)

0%	Deionized water	PBS	Mean
	4.65 ± 0.820	5.41 ± 0.760	5.03 ± 0.845^b^
25%	5.73 ± 0.438	6.34 ± 0.426	6.03 ± 0.520^a^
50%	6.48 ± 0.386	6.22 ± 0.492	6.35 ± 0.439^a^
75%	6.04 ± 0.263	6.34 ± 0.466	6.19 ± 0.390^a^
100%	4.67 ± 0.655	5.71 ± 0.470	5.19 ± 0.767^b^
Mean	5.51 ± 0.904^B^	6.00 ± 0.624^A^	

Different lowercase superscript letters in the Mean column indicate significant differences among cement formulations. Different uppercase superscript letters in the Mean row indicate a significant difference between storage media (Tukey’s test, *p* < 0.05). No letters were assigned to individual cement-by-medium combinations because the interaction was not statistically significant.

## Discussion

The present study evaluated the effect of incorporating different proportions of aspartic acid solution on the physicochemical properties and mineralization-related surface response of a calcium silicate-based hydraulic cement. The use of bioactive agents to modify hydraulic cements has been widely investigated as a strategy to enhance their properties and clinical performance [16, 21–23]. Aspartic acid contains functional groups capable of interacting with calcium and phosphate ions, thereby promoting mineral nucleation processes [6, 10]. In this context, the null hypothesis was rejected, as the incorporation of aspartic acid significantly influenced the evaluated properties.

Direct comparison with unmodified MTA demonstrated that the effects of aspartic acid were restricted to specific outcomes. The 25% formulation showed a shorter setting time and lower surface roughness, while the 25%, 50%, and 75% formulations exhibited higher Ap/C spectral ratios than the control. However, all modified formulations showed greater solubility and lower Ca²⁺ concentrations, and the higher proportions produced additional adverse effects on setting and surface roughness. Therefore, aspartic acid functionalization did not provide an overall improvement over unmodified MTA. Its potential added value was limited to the modulation of selected physicochemical and phosphate-associated spectral responses at specific proportions, and the biological or clinical relevance of these changes remains undetermined.

Setting time directly influences clinical handling and the material’s behavior in the biological environment [24]. The 25% formulation showed the shortest setting time, with significantly lower values than the control, 75%, and 100% formulations, but did not differ from the 50% group. The 50% formulation showed a setting time similar to that of the control but lower than those of the 75% and 100% groups. Although the 75% formulation showed longer setting times than the 25% and 50% formulations, it did not differ from the control. The 100% formulation exhibited the longest setting time and differed significantly from all other groups.

The concentration-dependent changes in setting time may be interpreted in the context of the dissolution–precipitation equilibria involved in calcium silicate hydration. Hydration releases Ca²⁺ and silicate species into the pore solution, followed by the precipitation of calcium silicate hydrate (C–S–H) and calcium hydroxide. Accumulation of Ca²⁺ and OH⁻ may reduce the driving force for further calcium silicate dissolution through a common-ion or product-inhibition effect, whereas removal of these species from solution by precipitation allows hydration to proceed [25–27]. At the lower aspartic acid proportions, partial coordination of Ca²⁺ by carboxylate groups may have reduced free calcium activity and alleviated this inhibition. The carboxylate groups may also have contributed to the initial organization of hydration nuclei. These combined effects could account for the shorter setting time observed particularly for the 25% formulation [26–30].

At higher aspartic acid proportions, however, more extensive calcium complexation and possible adsorption of organic species onto calcium-bearing surfaces may have delayed the attainment of supersaturation required for the precipitation and growth of C–S–H and Ca(OH)₂ [31]. Thus, although reducing free Ca²⁺ activity could favor continued calcium silicate dissolution by limiting the common-ion effect, excessive calcium sequestration may kinetically hinder the formation of the solid hydration products responsible for setting [25, 31]. The longer setting times observed at 75% relative to the 25% and 50% formulations, and particularly at 100% relative to all other groups, may therefore reflect a concentration-dependent balance among dissolution, product removal, calcium complexation, and precipitation kinetics rather than a conventional common-ion effect alone. Because early hydration kinetics and pore-solution speciation were not directly measured, this mechanistic interpretation remains hypothetical.

In addition to these effects, solubility plays an important role in the overall performance of the material. Excessive mass loss may compromise structural integrity and sealing ability, thereby negatively affecting clinical performance. According to ANSI/ADA guidelines, mass loss should not exceed 3% after immersion in deionized water for seven days. In the present study, all aspartic acid-containing formulations showed greater solubility than the control. The 25% formulation exhibited lower solubility than the 50%, 75%, and 100% formulations, whereas no significant differences were found among the three higher proportions. Therefore, aspartic acid increased cement solubility, although this effect was not progressive across all tested proportions. The 50%, 75%, and 100% formulations exceeded the recommended 3% limit.

These findings may reflect changes in hydration kinetics and matrix organization. Alterations in the nucleation and growth of hydration products can affect pore structure and susceptibility to ionic leaching [32, 33]. However, because cement porosity and matrix cohesion were not directly evaluated, these mechanisms should be regarded as possible explanations for the greater solubility rather than as demonstrated structural changes.

Negative solubility values observed in the 0% group indicate a net increase in specimen mass and have previously been reported for calcium silicate cements, including White MTA Angelus. This behavior may be attributed to the hydrophilic nature and ongoing hydration of these materials, which promote water uptake and retention within the cement structure and may outweigh mass loss caused by dissolution [34].

With regard to pH, a non-linear response was observed. Hydration of the calcium silicate phases begins after MTA is mixed with water and results in the formation of C–S–H and calcium hydroxide. Dissociation of calcium hydroxide releases hydroxyl ions and produces a highly alkaline environment, with the pH of freshly mixed MTA reported to increase during the first hours and approach approximately 12.5 [35]. This early alkalinization provides the expected baseline behavior of unmodified MTA.

In the present study, however, pH was not measured immediately after mixing or directly in the cement pore solution. It was measured once in the external deionized water after seven days of immersion. Therefore, the recorded values represent the alkalinizing effect maintained in the immersion medium at the end of this period rather than the maximum pH attained during early hydration. The control, 75%, and 100% formulations maintained pH values between 11.10 and 11.32, whereas the 25% and 50% formulations exhibited lower values. Because the pH of the aspartic acid stock solution was adjusted to 9.5 before cement mixing, this reduction cannot be attributed solely to the initial acidity of the mixing liquid. The ability of aspartate-containing molecules to interact with Ca²⁺ has been previously reported [3, 6]. Because calcium availability, hydration kinetics, and microstructure development are closely related in tricalcium silicate cements [27], interactions between deprotonated carboxylate groups and calcium may have modified calcium retention and the formation or dissolution of calcium-containing hydration products, thereby affecting hydroxyl ion availability. At the higher aspartic acid proportions, increased cement solubility may also have contributed to the release of alkaline species. Nevertheless, the lower endpoint pH values of the 25% and 50% formulations do not demonstrate that early calcium hydroxide formation or initial alkalinization was absent. Because pH and Ca²⁺ concentration were measured only after seven days, the initial pH peak, its temporal evolution, and the speciation of the released ions could not be determined.

The control group exhibited a higher Ca²⁺ concentration in the immersion solution than all aspartic acid-containing formulations. Calcium release is relevant to hydraulic calcium silicate cements because it contributes to an alkaline and calcium-rich environment and provides ionic species that may participate in the precipitation of calcium phosphate phases when phosphate is available [1]. In the present study, the higher Ca²⁺ concentration of the control was accompanied by lower solubility, suggesting that calcium release was not simply associated with greater matrix degradation. The lower Ca²⁺ concentrations of the modified formulations may instead reflect changes in cement hydration, dissolution, or calcium retention resulting from interactions with the carboxylate groups of aspartic acid.

Greater Ca²⁺ release, however, should not be considered advantageous in isolation. Excessive ionic leaching may be associated with increased porosity, water sorption, solubility, and loss of matrix stability [14]). Moreover, although the control exhibited the highest Ca²⁺ concentration, its Ap/C ratio was lower than those of the 25%, 50%, and 75% formulations. This finding indicates that calcium concentration in the immersion solution alone did not determine the phosphate-associated spectral response. Rather, the observed surface changes resulted from the combined influence of calcium availability, cement stability, aspartic acid interactions, and phosphate supplied by the storage medium. Thus, greater Ca²⁺ release may be favorable only when it occurs without compromising matrix stability and under environmental conditions that support calcium phosphate precipitation.

Together, these findings indicate that the effect of aspartic acid on the ionic environment was non-linear and may have reflected changes in calcium interactions, hydration kinetics, hydroxyl ion availability, and cement dissolution.

Surface roughness was significantly influenced by cement composition but not by the storage medium. The 25% formulation showed lower roughness than the control, 75%, and 100% formulations, whereas the 100% formulation exhibited the highest values. The intermediate behavior of the other formulations indicates that the effect of aspartic acid on surface topography was non-linear. These differences in Sa describe variations in surface height distribution but do not, by themselves, demonstrate changes in matrix homogeneity, porosity, or cohesion.

At 1000× magnification, SEM analysis showed qualitatively similar general surface morphologies among the experimental groups, without a consistent concentration-dependent pattern or readily distinguishable differences between storage media. Thus, the topographical differences detected by confocal microscopy were not accompanied by pronounced morphological changes detectable at the SEM magnification used. Fine-scale differences cannot be excluded because higher-magnification imaging and morphometric analysis of the deposits were not performed.

The absence of a significant storage-medium effect on Sa indicates that deionized water and PBS did not produce statistically different roughness values under the conditions evaluated. This result does not exclude medium-dependent changes in the chemical composition of the surface deposits, which were assessed by EDS and ATR-FTIR.

ATR-FTIR analysis revealed phosphate- and carbonate-associated spectral bands, but their interpretation depended on the storage medium. EDS detected negligible phosphorus in specimens stored in deionized water, confirming the suitability of this medium as a phosphate-free control and indicating that the phosphate-associated FTIR bands alone should not be interpreted as evidence of relevant calcium phosphate deposition under this condition. In contrast, the marked phosphorus incorporation detected after PBS storage supported the presence of Ca–P-rich surface deposits and demonstrated the dependence of this response on phosphate supplied by the external medium. The combined findings were compatible with an apatite-like surface phase in PBS-stored specimens, but neither EDS nor FTIR provided definitive identification of crystalline apatite.

Among specimens stored in PBS, the intensity of phosphate-associated bands varied according to the proportion of aspartic acid. The 25%, 50%, and 75% formulations exhibited higher phosphate-associated spectral signals than the control and 100% formulations. This pattern was also observed in the Ap/C analysis, in which the intermediate formulations showed significantly higher ratios than the 0% and 100% groups. However, the Ap/C ratio represents the relative contribution of phosphate-associated bands to carbonate-associated bands and does not quantify the amount of mineral deposited or confirm apatite formation. Moreover, EDS did not demonstrate a consistent concentration-dependent increase in phosphorus content. Therefore, these findings indicate differences in the relative spectral composition of the surface deposits rather than enhanced biomineralization or optimal mineral formation.

The two-way ANOVA demonstrated a significant main effect of storage medium. When averaged across cement formulations, specimens stored in PBS exhibited higher marginal Ap/C values than those stored in deionized water. However, the cement formulation-by-storage medium interaction was not significant, indicating no statistical evidence that the effect of the storage medium differed among formulations. These findings indicate that phosphate availability influenced the overall Ap/C spectral response, although individual group means did not consistently follow the same numerical direction. This overall effect is consistent with previous studies showing that phosphate-containing media favor the precipitation of Ca–P-rich surface deposits on hydraulic cements [18, 29].

From a mechanistic perspective, these findings are consistent with the possibility that aspartic acid influenced the phosphate-associated surface response through interactions between its carboxylate groups and calcium ions, as previously reported for non-collagenous protein analogues [3, 6, 11]. However, the negligible surface phosphorus content detected after storage in deionized water indicates that any effect associated with aspartic acid was insufficient to produce Ca–P-rich deposits in the absence of an external phosphate source.

Taken together, aspartic acid functionalization produced outcome-specific effects rather than a uniform improvement over unmodified MTA. The 25% formulation showed shorter setting time and lower surface roughness, whereas the intermediate formulations exhibited higher Ap/C spectral ratios. These changes were accompanied by greater solubility and lower Ca²⁺ concentrations in all modified formulations, and the 100% formulation showed unfavorable changes in setting time and surface roughness. Therefore, the potential benefit of aspartic acid was restricted to specific properties and proportions, and no formulation improved all evaluated outcomes.

The absence of crystallographic characterization and high-magnification imaging of the surface deposits represents a limitation of this study. SEM analysis at 1000× allowed a qualitative comparison of general surface organization and deposit distribution but did not permit detailed assessment of individual deposits or nanoscale structures. Therefore, the absence of readily distinguishable morphological patterns at this magnification does not exclude differences at finer scales. In PBS-stored specimens, the combined EDS and FTIR findings supported the presence of Ca–P-rich surface deposits with spectral features compatible with an apatite-like phase. However, these methods do not provide definitive identification of a crystalline phase. Conventional XRD may also have limited sensitivity for thin or poorly crystalline surface layers because the diffraction pattern can be dominated by the underlying cement matrix. Future studies should combine surface-sensitive crystallographic techniques, such as grazing-incidence XRD, with higher-magnification SEM or field-emission SEM to characterize the crystalline nature and detailed morphology of these deposits. Further investigations of cement hydration, microstructure, phase stability, mechanical properties, and biological response are also required to determine the potential applicability of aspartic acid functionalization.

## Conclusion

The incorporation of aspartic acid produced non-linear and formulation-dependent changes in the physicochemical properties and phosphate-associated surface response of the hydraulic cement. Compared with the control, the 25% formulation reduced setting time and surface roughness. However, all aspartic acid-containing formulations exhibited greater solubility and lower Ca²⁺ concentrations in the immersion solution, with the 50%, 75%, and 100% formulations exceeding the recommended solubility limit. The intermediate formulations exhibited higher Ap/C spectral ratios than the 0% and 100% formulations, indicating a greater relative contribution of phosphate-associated FTIR bands to carbonate-associated bands. This finding does not demonstrate increased apatite formation or confirm the presence of a crystalline apatite phase. EDS findings indicated that Ca–P-rich surface deposition depended on phosphate supplied by the storage medium. The 100% formulation prolonged setting time and produced the highest surface roughness. Therefore, no aspartic acid proportion improved all evaluated properties, and further physicochemical and biological investigations are required before the potential applicability of this approach can be established.

## Data Availability

The datasets generated and analyzed during the current study are not publicly available but are available from the corresponding author upon reasonable request.
